# The Transverse Bearing Characteristics of the Pile Foundation in a Calcareous Sand Area

**DOI:** 10.3390/ma15176176

**Published:** 2022-09-05

**Authors:** Haibo Hu, Lina Luo, Gang Lei, Jin Guo, Shaoheng He, Xunjian Hu, Panpan Guo, Xiaonan Gong

**Affiliations:** 1 Research Center of Coastal and Urban Geotechnical Engineering, Zhejiang University, Hangzhou 310058, China; 2 College of Railway Engineering, Guangzhou Railway Polytechnic, Guangzhou 510430, China; 3 Beijing Urban Construction Design & Development Group Company Limited, Beijing 100037, China

**Keywords:** calcareous sand, pile foundation, transverse bearing characteristics, Pasternak foundation model, theory method

## Abstract

Reviewing literature revealed that the studies on the bearing characteristics of pile foundations mainly focuses on clay, ordinary sand, loess, saline soil, and other areas. However, few studies on the bearing characteristics of the pile foundation in calcareous sand were conducted. Besides, existing traditional studies ignored the variation of soil compression modulus with depth, and the effect of void ratio on the transverse bearing characteristics of the pile foundation in a calcareous sand area were not well understood. In response of these problems, this study conducted a theoretical investigation on the transverse bearing characteristics of the pile foundation in a calcareous sand area. The transverse bearing characteristics of the pile foundation were derived based on the Pasternak foundation model and the Winkler foundation model, incorporating the heterogeneous distribution of compressive modulus with buried depth. The calculation results of the Pasternak foundation model are closer to the observed results than the Winkler foundation model. Therefore, the following research on the transverse bearing characteristics of the pile foundation in the calcareous sand area adopts the Pasternak foundation model. Then, the effects of the pile length, pile diameter, pile elastic modulus, horizontal load, bending moment, and void ratio on the transverse bearing characteristics of the pile foundation in a calcareous sand area were thoroughly analyzed. Furthermore, the difference between the transverse bearing characteristics of the pile foundation in a calcareous sand area and a quartz sand area was discussed. Results show that the horizontal displacement of the pile top in a calcareous sand area is greater than the quartz sand area under the same conditions.

## 1. Introduction

With the development of engineering construction, horizontally loaded piles have been widely used [1,2,3,4]. To investigate the behavior of horizontally loaded piles, the methods of finite element, elasticity theory, and elastic foundation reaction are commonly adopted. The finite element method has a wide range of applications. However, in some cases, the finite element analysis process is troublesome, hindering it from solving practical engineering problems [5,6,7]. Poulos (1971) established the elastic analysis method of horizontally loaded piles by using Mindlin elastic solution [8], but this method is mainly applicable to a homogeneous foundation. Compared to the finite element method and elasticity theory method, the elastic foundation reaction method based on the Winkler foundation model has a clear concept, a simple solution process, and is thus more practical in engineering [9]. The elastic foundation reaction method is also extensively employed in the two parameter foundation models, capable of considering the shear action between soil springs, including the Pasternak foundation model and the Vlasov foundation model [10,11].

Calcareous sand is a special sand material formed by the transportation and accumulation of corals and other marine life debris (such as seaweed, shells, etc.) by seawater. Its main chemical composition is calcium carbonate, and it is distributed extensively in low latitude sea areas, such as the South China Sea [12,13,14,15]. Most of the construction of marine reclamation islands and reefs are far away from the land, the resources of filling materials on the islands are limited, and the cost of transporting fillers from land is relatively high. Therefore, many island and reef projects around the world use local materials and use the coral reef calcium distributed on the island reefs. As the backfill of the foundation, the calcareous sand can not only shorten the construction period but also save landfill resources, which is of great significance for the development of the modern marine economy [16]. Reviewing literature revealed that the studies on the bearing characteristics of pile foundation mainly focuses on clay [17,18,19], ordinary sand [20,21,22], loess [23], saline soil [24]. and other areas [25]. However, few studies on the bearing characteristics of the pile foundation in calcareous sand were conducted. With the rise of pile foundation construction in calcareous sand areas, the transverse static characteristics of the pile foundation needs to be urgently studied. The change of soil compression modulus with depth and void ratio is recognized as a key issue in the theoretical calculation of foundation engineering in calcareous sand areas. However, on the whole, the above-mentioned research lacks consideration of the variation of compressive modulus with the buried depth and void ratio. In addition, there has been a gap in understanding the differences in the transverse bearing characteristics of the pile foundation in calcareous sand areas and quartz sand areas under different factors.

Considering the change of the compressive modulus of the calcareous sand with confining pressure, this study investigated the transverse bearing characteristics of the pile foundation in the calcareous sand area by the Pasternak foundation model. In addition, the effects of the pile length, pile diameter, pile elastic modulus, horizontal load, bending moment, and void ratio on the transverse bearing characteristics is thoroughly investigated. Furthermore, the differences between the transverse characteristics of the pile foundation in the calcareous sand area and quartz sand area are deeply discussed.

## 2. Methodology

### 2.1. Pile Foundation Response Analysis Based on Pasternak Foundation Model

**Figure 1:**
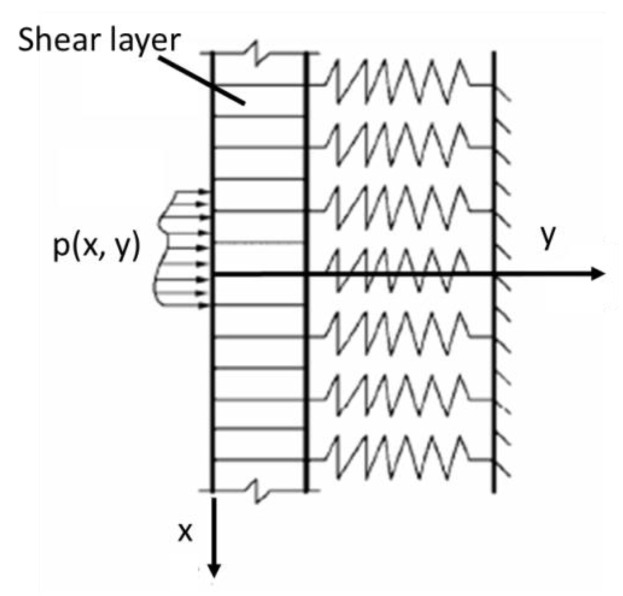
Pasternak foundation model.

Based on the Winkler foundation model, the Pasternak foundation model assumes that there is a shear layer on the spring element, and this layer can only produce shear deformation but not compressible deformation, so that there is shear interaction between the spring elements, as shown in Figure 1.

The differential equation of lateral deformation of pile on the Pasternak foundation is:

(1) $$E_{p}I_{p}\frac{d^{4}y}{{\mathrm{dz}}^{4}}-GD_{e}\frac{d^{2}y}{{\mathrm{dz}}^{2}}+Ky=0$$

where *E*_p_*I*_p_ (kN·m^2^) and *D*_e_ (m) are the bending stiffness and equivalent width of the pile, respectively; y is the pile deflection (m); K (kN/m^2^) and G (kN/m) are two parameters of the Pasternak foundation, namely foundation reaction modulus and shear layer stiffness.

For the shear layer stiffness G, the empirical formula proposed by Tanahashi and Hideaki (2004) is adopted [26]:

(2) $$G=\frac{E_{s}t}{6\left(1-\nu \right)}$$

where $E_{s}$ is the elastic modulus of the foundation soil (kPa); *t* is the thickness of the shear layer (m). According to the finite element results of Yao and Yin (2010) [27], *t* = 11 d is taken, and *D* is the diameter of pile foundation; ν is the Poisson’s ratio of the foundation soil.

For the selection of foundation reaction modulus K, the calculation method proposed by Vesic et al. has been mostly used in previous studies [28,29,30]. However, the premise of this formula is that the elastic foundation beam is placed on the surface of the elastic half space, leading to the fact that the embedded depth of the beam cannot be considered. Therefore, the calculation formula of Yu et al., (2013) considering the buried depth was adopted [31]:

(3) $$K=\frac{3.08E_{s}}{\eta \left(1-\nu^{2}\right)}{\left[\frac{E_{s}D_{e}{}^{4}}{E_{p}I_{p}}\right]}^{1/8}$$

(4) $$\eta =\left\{ \begin{array}{l} 2.18\;\;\;\;\;,h/D_{e}\leq 0.5\\ \left(1+\frac{1}{1.7h/D_{e}}\right),h/D_{e}>0.5 \end{array}\right.$$

where η is the depth parameter, and *h* is the buried depth of the pile (m).

**Figure 2:**
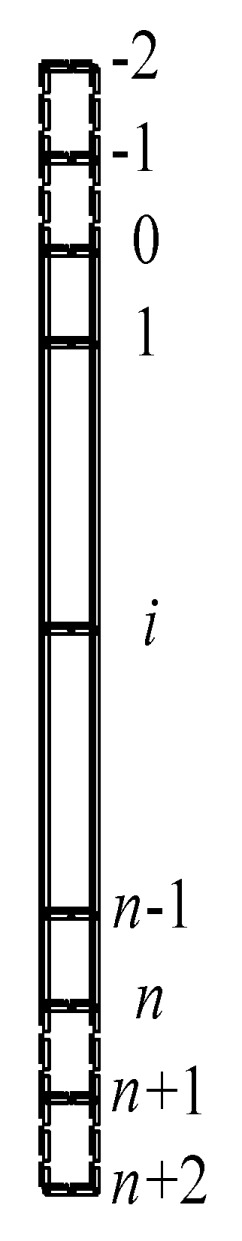
Discrete analysis model of pile.

The fourth-order differential equation is solved by the finite difference method. The node element is shown in Figure 2.

The pile is divided into *n* equal parts, where each length is denoted as *l*. Two virtual nodes are added at both ends of the pile, and the total number of nodes is *n* + 5. Then, Equation (1) can be converted into the finite difference format:

(5) $$E_{p}I_{p}\frac{6y_{i}-4\left(y_{i+1}+y_{i-1}\right)+\left(y_{i+2}+y_{i-2}\right)}{l^{4}}-GD_{e}\frac{y_{i+1}-2y_{i}+y_{i-1}}{l^{2}}+Ky_{i}=0$$

In order to ensure the calculation accuracy, the length of the pile part is separated into 0.05 m in the calculation of this article, and the loading direction is horizontal to the right on the top of the pile.

Given the boundary conditions—the external force and bending moment act on the pile top, and the pile top is free—the following equations can be obtained:


(6) $$\left\{\begin{array}{c} M_{0}=-E_{p}I_{p}\frac{d^{2}y}{\;dz^{2}}=-{\left.E_{p}I_{p}\frac{y_{i+1}-2y_{i}+y_{i-1}}{l^{2}}\right|}_{i=0}=M\\ M_{n}=-E_{p}I_{p}\frac{d^{2}y}{\;dz^{2}}=-{\left.E_{p}I_{p}\frac{y_{i+1}-2y_{i}+y_{i-1}}{l^{2}}\right|}_{i=n}=0\\ Q_{0}=-E_{p}I_{p}\frac{d^{3}y}{\;dz^{3}}=-{\left.E_{p}I_{p}\frac{y_{i+2}-2y_{i+1}+2y_{i-1}-y_{i-2}}{2l^{3}}\right|}_{i=0}=H\\ Q_{n}=-E_{p}I_{p}\frac{d^{3}y}{\;dz^{3}}=-{\left.E_{p}I_{p}\frac{y_{i+2}-2y_{i+1}+2y_{i-1}-y_{i-2}}{2l^{3}}\right|}_{i=n}=0 \end{array}\right.$$

Substitute the expression obtained from Equation (6) into Equation (5), and write it as a matrix expression:

(7) $$\left(K_{1}-K_{2}+K_{3}\right)y=F$$

where $K_{1}$ is the pile deformation stiffness matrix, $K_{2}$ is the foundation shear stiffness matrix, $K_{3}$ is the foundation stiffness matrix, and F is the column vector of external load. The matrix expressions are as follows:

(8) $$\;K_{1}=\frac{E_{p}I_{p}}{l^{4}}{\left[\begin{array}{ccccccc} 2 & -4 & 2 & & & & \\ -2 & 5 & -4 & 1 & & 0 & \\ 1 & -4 & 6 & -4 & 1 & & \\ & \ddots & \ddots & \ddots & \ddots & \ddots & \\ & & 1 & -4 & 6 & -4 & 1\\ & 0 & & 1 & -4 & 5 & -2\\ & & & & 2 & -4 & 2 \end{array}\right]}_{\left(n+1\right)\left(n+1\right)}$$

(9) $$K_{2}=\frac{GD_{e}}{l^{2}}{\left[\begin{array}{ccccccc} 0 & & & & & & \\ 1 & -2 & 1 & & & 0 & \\ & 1 & -2 & 1 & & & \\ & \ddots & \ddots & \ddots & \ddots & \ddots & \\ & & & 1 & -2 & 1 & \\ & 0 & & & 1 & -2 & 1\\ & & & & & & 0 \end{array}\right]}_{\left(n+1\right)\left(n+1\right)}$$

(10) $$\;K_{3}=K{\left[\begin{array}{ccccccc} 1 & & & & & & \\ & 1 & & & & 0 & \\ & & 1 & & & & \\ & \ddots & \ddots & \ddots & \ddots & \ddots & \\ & & & & 1 & & \\ & 0 & & & & 1 & \\ & & & & & & 1 \end{array}\right]}_{\left(n+1\right)\left(n+1\right)}$$

(11) $$y={\left[\begin{array}{ccccccc} y_{0}, & y_{1}, & y_{2}, & \cdots , & y_{n-2}, & y_{n-1}, & y_{n} \end{array}\right]}^{T}{}_{\left(n+1\right)}$$

(12) $$F={\left[\begin{array}{ccccc} -\frac{2M+2Hl}{l^{2}}-\frac{GD_{e}M}{EI}, & \frac{M}{l^{2}}, & 0, & \cdots , & 0 \end{array}\right]}^{T}{}_{\left(n+1\right)}$$

### 2.2. Pile Foundation Response Analysis Based on Winkler Foundation Model

The differential equation of lateral deformation of the pile on Winkler foundation is:

(13) $$E_{p}I_{p}\frac{d^{4}y}{{\mathrm{dz}}^{4}}+Ky=0$$

where *E*_p_*I*_p_ and *D*_e_ are the bending stiffness and equivalent width of the pile, respectively; y is the pile deflection; the selection of the foundation reaction modulus K is consistent with the above Pasternak foundation model.

The fourth-order differential equation is also obtained by the finite difference method:

(14) $$E_{p}I_{p}\frac{6y_{i}-4\left(y_{i+1}+y_{i-1}\right)+\left(y_{i+2}+y_{i-2}\right)}{l^{4}}+Ky_{i}=0$$

Using the same boundary conditions as the Equation (6), the following equation is given by:


(15) $$\left\{\begin{array}{c} M_{0}=-E_{p}I_{p}\frac{d^{2}y}{\;dz^{2}}=-{\left.E_{p}I_{p}\frac{y_{i+1}-2y_{i}+y_{i-1}}{l^{2}}\right|}_{i=0}=M\\ M_{n}=-E_{p}I_{p}\frac{d^{2}y}{\;dz^{2}}=-{\left.E_{p}I_{p}\frac{y_{i+1}-2y_{i}+y_{i-1}}{l^{2}}\right|}_{i=n}=0\\ Q_{0}=-E_{p}I_{p}\frac{d^{3}y}{\;dz^{3}}=-{\left.E_{p}I_{p}\frac{y_{i+2}-2y_{i+1}+2y_{i-1}-y_{i-2}}{2l^{3}}\right|}_{i=0}=H\\ Q_{n}=-E_{p}I_{p}\frac{d^{3}y}{\;dz^{3}}=-{\left.E_{p}I_{p}\frac{y_{i+2}-2y_{i+1}+2y_{i-1}-y_{i-2}}{2l^{3}}\right|}_{i=n}=0 \end{array}\right.$$

Substitute the expression obtained from Equation (15) into Equation (14), and write it as a matrix expression:

(16) $$\left(K_{a}+K_{b}\right)y=P$$

where $K_{a}$ is the pile deformation stiffness matrix, $K_{b}$ is the foundation stiffness matrix, and v is the column vector of the external load. The matrix expressions are as follows:

(17) $$\;K_{a}=\frac{E_{p}I_{p}}{l^{4}}{\left[\begin{array}{ccccccc} 2 & -4 & 2 & & & & \\ -2 & 5 & -4 & 1 & & 0 & \\ 1 & -4 & 6 & -4 & 1 & & \\ & \ddots & \ddots & \ddots & \ddots & \ddots & \\ & & 1 & -4 & 6 & -4 & 1\\ & 0 & & 1 & -4 & 5 & -2\\ & & & & 2 & -4 & 2 \end{array}\right]}_{\left(n+1\right)\left(n+1\right)}$$

(18) $$\;K_{b}=K{\left[\begin{array}{ccccccc} 1 & & & & & & \\ & 1 & & & & 0 & \\ & & 1 & & & & \\ & \ddots & \ddots & \ddots & \ddots & \ddots & \\ & & & & 1 & & \\ & 0 & & & & 1 & \\ & & & & & & 1 \end{array}\right]}_{\left(n+1\right)\left(n+1\right)}$$

(19) $$y={\left[\begin{array}{ccccccc} y_{0}, & y_{1}, & y_{2}, & \cdots , & y_{n-2}, & y_{n-1}, & y_{n} \end{array}\right]}^{T}{}_{\left(n+1\right)}$$

(20) $$P={\left[\begin{array}{ccccc} -\frac{2M+2Hl}{l^{2}}, & \frac{M}{l^{2}}, & 0, & \cdots , & 0 \end{array}\right]}^{T}{}_{\left(n+1\right)}$$

### 2.3. Case Verification

Filho et al. (2005) verified the horizontally loaded single pile test of Kerisel and Adam (1967) by using the finite element method [32,33]. The pile length of the pile foundation is 4.65 m, the pile diameter is 0.36 m, and the elastic modulus is 20 GPa. The pile top is subjected to a horizontal load of 60 kN and a bending moment of 69 kN·m. The pile top is free. The elastic modulus of the soil is 9.233 MPa, and Poisson’s ratio is 0.3.

**Figure 3:**
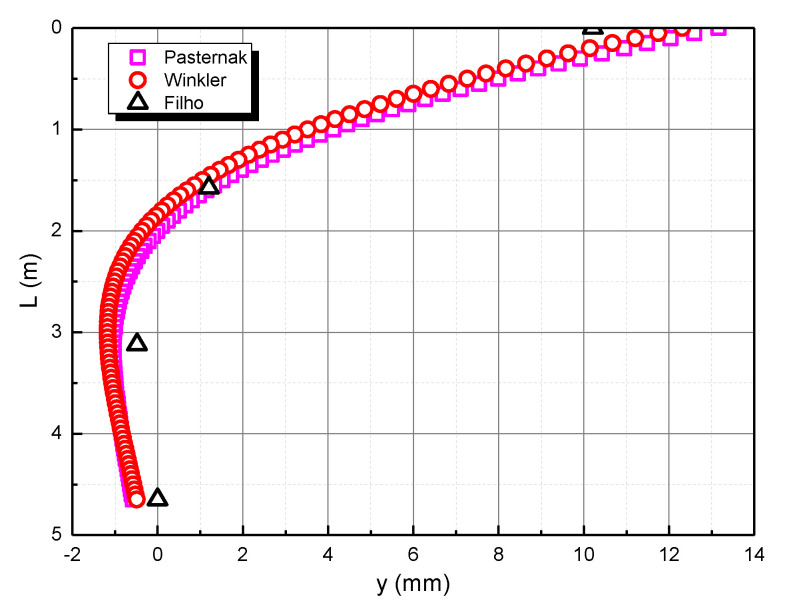
Comparison of horizontal displacement of pile body.

Figure 3 shows the comparison between the calculation method of this study and the Filho method. The calculation results of the Pasternak foundation model are closer to the observed results than the Winkler foundation model. Therefore, the following research on the transverse bearing characteristics of the pile foundation in the calcareous sand area will adopt the Pasternak foundation model.

## 3. Results

**Figure 4:**
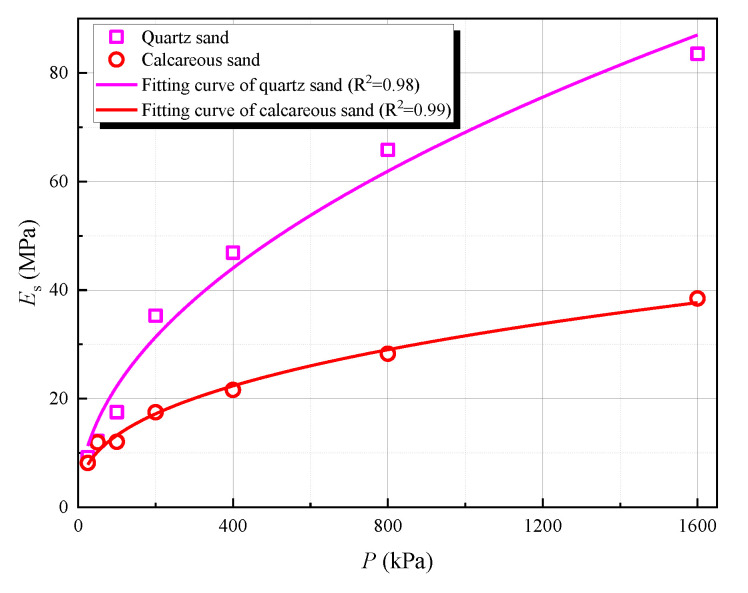
Correlation between compressive modulus and confining pressure.

In actual engineering, the compression modulus of soils is enhanced with the increase of buried depth. However, the above-mentioned Pasternak foundation model did not consider the change of soil compression modulus with buried depth, hindering a thorough understanding of the transverse bearing characteristics of the pile foundation. Through the triaxial tests of calcareous sand, a certain correlation between calcareous sand compression modulus and confining pressure was observed, as shown in Figure 4.

In this experiment, the weight of calcareous sand is 13.8 kN/m^3^, and the static earth pressure coefficient is 0.5. Through insight curve fitting, the relationship between the compressive modulus and the confining pressure of calcareous sand can be obtained as follows:

(21) $$E_{s}=0.1314\times 101.4\times {\left(P_{i}/101.4\right)}^{0.3768}=\frac{0.262}{1+e^{3}}\times 101.4\times {\left(6.9h/101.4\right)}^{0.3768}$$

where $E_{s}$ is the compression modulus of calcareous sand (MPa); $P_{i}$ is the confining pressure of calcareous sand (kPa); e is the void ratio of calcareous sand, which is 0.998; h is buried depth.

**Figure 5:**
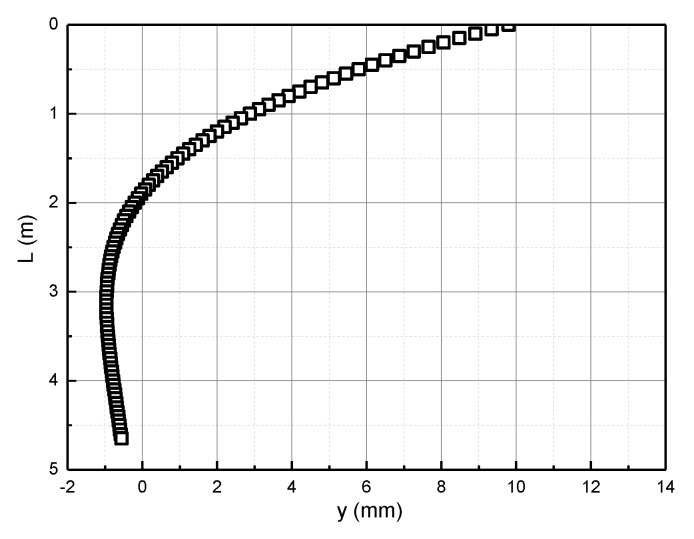
Horizontal displacement of pile body.

The obtained compression modulus $E_{s}$ is substituted into the Pasternak foundation model, and then the horizontal deformation curve of the pile foundation is obtained according to MATLAB programming. According to the pile foundation size and load in the calculation example verification, the distribution of the horizontal deformation of the pile body along the pile length is obtained, as shown in Figure 5.

Based on the engineering conditions of the above example, the effects of the pile length, pile diameter, pile elastic modulus, horizontal load, bending moment, and void ratio on the horizontal displacement of the pile body in the calcareous sand area are further analyzed.

During the following analysis, the pile length of the pile foundation (L) is 4.65 m, the pile diameter (D) is 0.36 m, and the elastic modulus (E) is 20 GPa. The horizontal load (H) is 60 kN, and the bending moment (M) is 69 kN·m. The pile top is free. The compressive modulus is valued according to Equation (21) and Equation (22), and Poisson’s ratio is 0.3.

### 3.1. Effect of Pile Length

**Figure 6:**
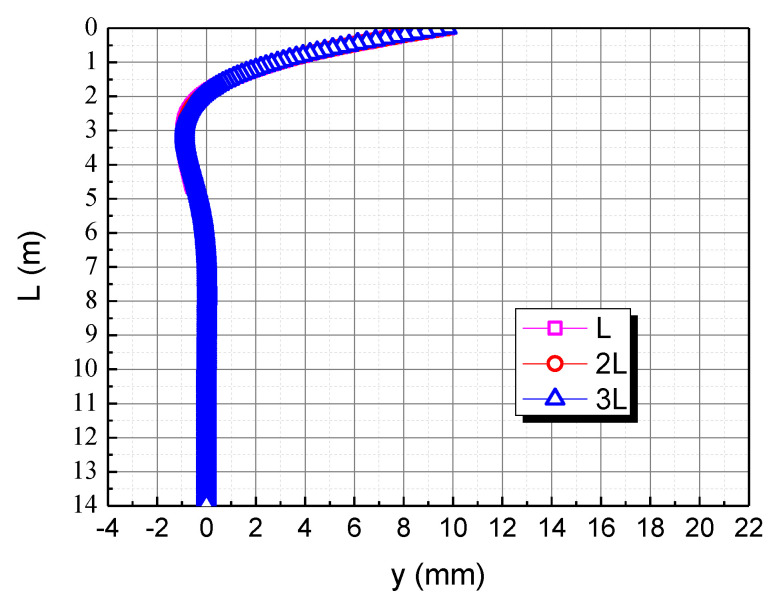
Effect of pile length on horizontal displacement of pile body in calcareous sand area.

The pile length is taken as L, 2L, and 3L, respectively, to assess the effect of pile length on the horizontal deformation in the calcareous sand area, as shown in Figure 6. As can be seen, the horizontal displacement of the pile body of piles almost coincides, and the maximum displacement is near the pile top; there is a certain negative displacement near 1.9 m~6.6 m, and the horizontal displacement of the pile body beyond 6.6 m is almost 0. Therefore, it is evident that the horizontal load and the bending moment have the most obvious effect on the pile top. However, after exceeding a certain depth, these will no longer affect the horizontal displacement of the pile body. The main reason is that when the burial depth is large, the calcareous sand restricts the displacement of the pile body, so the displacement is almost 0. The application of the pile top load makes the pile top displacement larger. Due to the bottom of the pile being constrained, negative displacement occurs at a certain position below the pile top.

### 3.2. Effect of Pile Diameter

**Figure 7:**
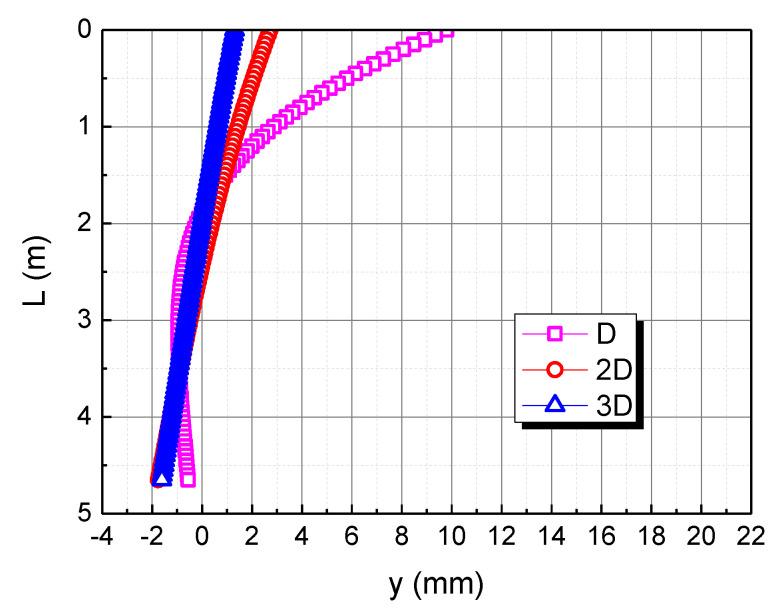
Effect of pile diameter on horizontal displacement of pile body in calcareous sand area.

The pile diameter is taken as D, 2D, and 3D, respectively, to check the effect of the pile diameter on the horizontal deformation in the calcareous sand area, as shown in Figure 7. As the pile diameter increases, the horizontal displacement of the pile top decreases. The rigid rotation of the pile body is caused by the action of the horizontal load and the bending moment. When the pile diameter is 2D and 3D, the horizontal displacement curve of the pile body is almost straight because of the increase in the pile body’s bending stiffness, resulting from the increase of the pile diameter. When the pile diameter is small, the stiffness of the pile body is small, and the pile body is more prone to deflection and deformation under the action of the pile top load. With the continuous increase of the pile diameter, the stiffness of the pile body increases, and the pile body is not prone to deflection, so the deformation is closer to a straight line.

### 3.3. Effect of Pile Elastic Modulus

**Figure 8:**
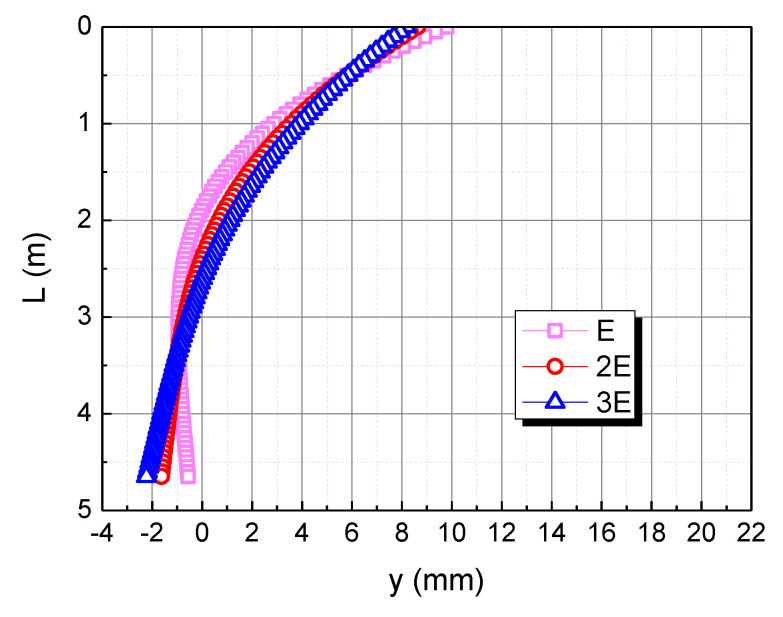
Effect of elastic modulus of pile on horizontal displacement of pile in calcareous sand area.

The elastic modulus of the pile body is taken as E, 2E, and 3E, respectively, to understand the effect of the pile elastic modulus on the horizontal deformation in the calcareous sand area, as shown in Figure 8. As the elastic modulus of the pile body increases, the horizontal displacement of the pile top decreases. When the elastic modulus of the pile body is 2E and 3E, the horizontal displacement curve of the pile body tends to be a straight line. This is because the bending stiffness of the pile body increases with the increase of the elastic modulus of the pile body, and the pile body rotates rigidly due to the action of the horizontal load and the bending moment.

### 3.4. Effect of Horizontal Load

**Figure 9:**
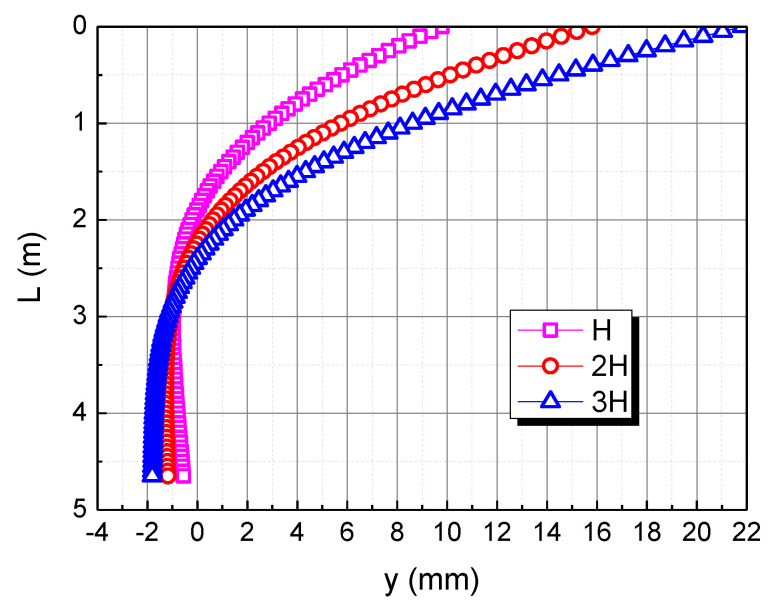
Effect of horizontal load on horizontal displacement of pile in calcareous sand area.

To analyze the effect of the horizontal load, the horizontal load is taken as H, 2H, and 3H, respectively. The influence of the horizontal load on the horizontal displacement of pile body in the calcareous sand area is shown in Figure 9. As the horizontal load increases, the horizontal displacement of the pile top increases. When the horizontal load is 2H and 3H, the horizontal displacement of the pile top is 15.8 mm and 21.8 mm, respectively. The horizontal displacement of the three piles is 0 when the depth is 1.9 m, 2.2 m, and 2.4 m, respectively, indicating that the greater the horizontal load, the deeper the 0 point of the pile displacement. This is mainly because the greater the horizontal load of the pile top, the greater the horizontal displacement of the pile top, which makes the pile as a whole more inclined to the load direction, so the 0 point of the pile displacement is deeper.

### 3.5. Effect of Bending Moment

**Figure 10:**
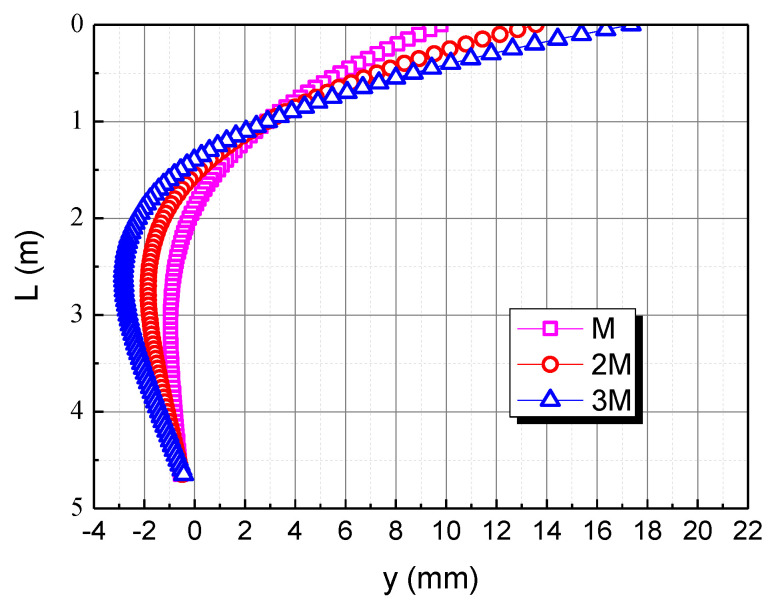
Effect of bending moment on horizontal displacement of pile in calcareous sand area.

To reveal the effect of the bending moment, the bending moment is taken as M, 2M, and 3M, respectively. The influence of the bending moment on the horizontal displacement of the pile body in the calcareous sand area is shown in Figure 10. As the bending moment increases, the horizontal displacement of the pile top increases. When the bending moment is 2M and 3M, the horizontal displacement of the pile top is 13.6 mm and 17.4 mm, respectively. The horizontal displacement of the three piles is 0 at the depth of 1.9 m, 1.5 m and 1.4 m respectively, which indicates that the greater the bending moment, the shallower the 0 point of the pile displacement. This is mainly due to the fact that the greater the bending moment, the greater the deflection of the pile, which makes the 0 point of the pile displacement shallower.

### 3.6. Effect of Void Ratio

**Figure 11:**
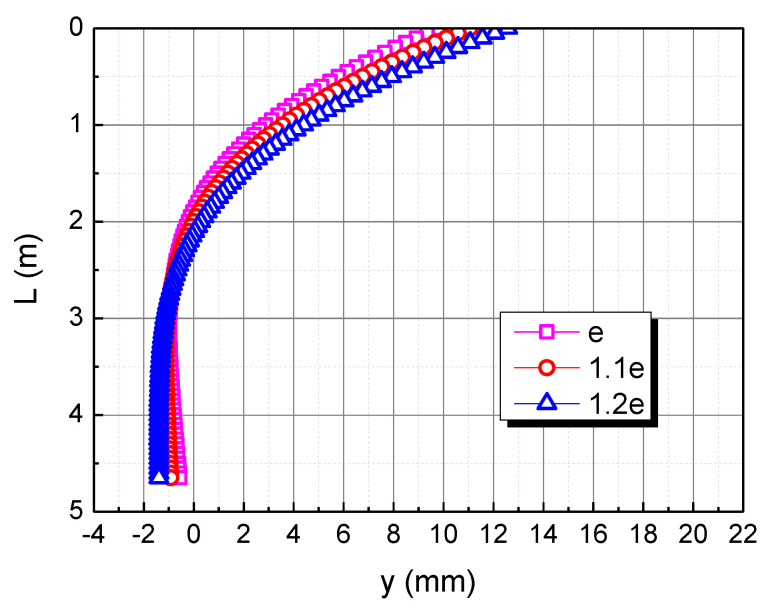
Effect of void ratio on horizontal displacement of pile in calcareous sand area.

The adopted void ratio (e) is taken as 0.998, corresponding to a relative density of 80%. To examine the effect of the void ratio, the 1.1e and 1.2e were also employed in the analysis, they did not exceed the maximum void ratio of the test of calcareous sand. The influence of the void ratio on the horizontal displacement of the pile body in the calcareous sand area is shown in Figure 11. As the void ratio increases, the horizontal displacement of the pile top increases. When the bending moment is 1.1e and 1.2e, the horizontal displacement of pile top is 11.1 mm and 12.6 mm, respectively. The horizontal displacement of the three piles is 0 at the depth of 1.9 m, 2 m, and 2.1 m respectively, which indicates that the greater the void ratio, the deeper the 0 point of the pile displacement. This is mainly due to the fact that the greater the void ratio, the easier the calcareous sand is to be compressed, and the pile as a whole is inclined to the load direction, making the 0 point of the pile displacement deeper.

## 4. Discussion

Moreover, to deeply analyze the pile behavior in the calcareous sand area under various influencing factors, the comparable study of the transverse bearing characteristics of the pile foundation in the calcareous sand and quartz sand area was conducted.

Through the triaxial tests of quartz sand, a certain correlation between quartz sand compression modulus and confining pressure was also observed, as shown in Figure 4. In this experiment, the weight of quartz sand is 18.2 kN/m^3^, and the static earth pressure coefficient is 0.5. Through insight curve fitting, the relationship between the compressive modulus and the confining pressure of quartz sand can be obtained as follows:

(22) $$E_{sq}=0.2215\times 101.4\times {\left(P_{iq}/101.4\right)}^{0.4906}=\frac{0.236}{1+e^{3}}\times 101.4\times {\left(9.1h/101.4\right)}^{0.4906}$$

where $E_{sq}$ is the compression modulus of quartz sand (MPa); $P_{iq}$ is the confining pressure of quartz sand (kPa); e is the void ratio of quartz sand, which is 0.4; h is buried depth.

### 4.1. Comparison of Pile Length in Calcareous Sand and Quartz Sand Area

**Figure 12:**
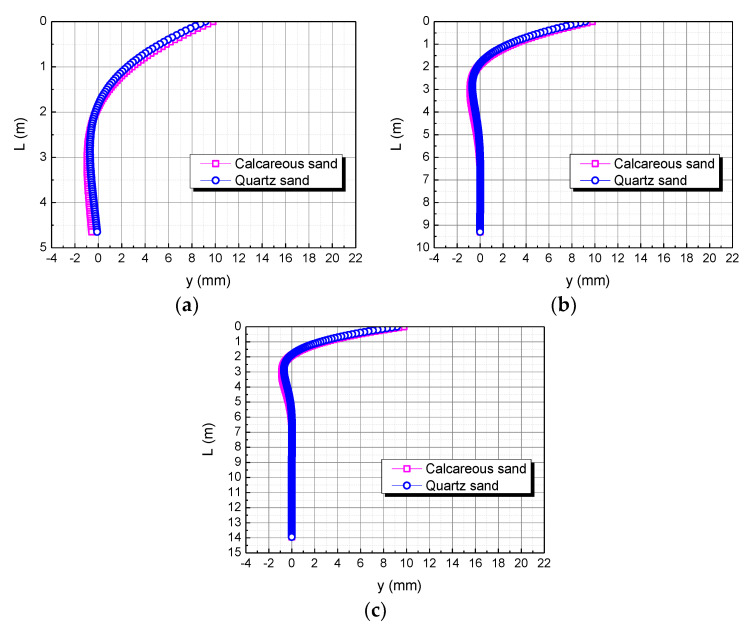
Effect of pile length on horizontal displacement of pile body. (**a**) L; (**b**) 2L; (**c**) 3L.

The pile length is taken as L, 2L, and 3L, respectively. The influence of the pile length on the horizontal displacement of the pile body is shown in Figure 12. When the pile length is L, the horizontal displacement of the pile top in the calcareous sand area is 0.63122 mm larger than in the quartz sand area. When the pile length is 2L, the horizontal displacement of the pile top in the calcareous sand area is 0.60970 mm larger than in the quartz sand area. When the pile length is 3L, the horizontal displacement of the pile top in the calcareous sand area is 0.60969 mm larger than in the quartz sand area. The smaller the pile length, the greater the difference between the two. It may be because the smaller the pile length, the greater the stiffness of the pile body, which makes calcareous sand easier to compress than quartz sand.

### 4.2. Comparison of Pile Diameter in Calcareous Sand and Quartz Sand Area

**Figure 13:**
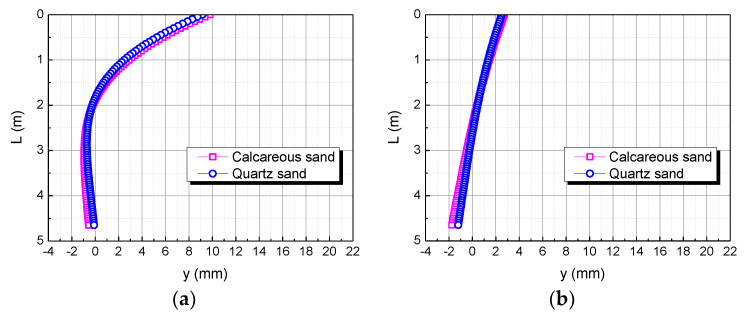

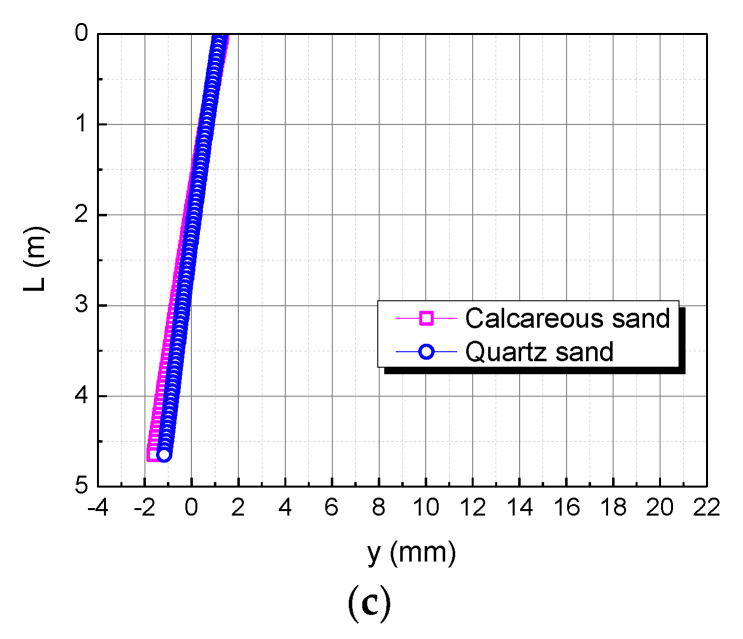
Effect of pile diameter on horizontal displacement of pile body. (**a**) D; (**b**) 2D; (**c**) 3D.

The pile diameter is taken as D, 2D, and 3D, respectively. The influence of the pile diameter on the horizontal displacement of the pile body is shown in Figure 13. When the pile diameter is D, the horizontal displacement of the pile top in the calcareous sand area is 0.63122 mm larger than in the quartz sand area. When the pile length is 2D, the horizontal displacement of the pile top in the calcareous sand area is 0.21888 mm larger than in the quartz sand area. When the pile length is 3D, the horizontal displacement of the pile top in the calcareous sand area is 0.07437 mm larger than in the quartz sand area. The larger the pile diameter, the smaller the difference between the two. This may be because the larger the pile diameter, the larger the contact area between the pile body and the soil body, and the smaller the stress on the soil body under the same area, which makes the difference in the displacement of the pile top under the conditions of calcareous sand and quartz sand smaller.

### 4.3. Comparison of Pile Elastic Modulus in Calcareous Sand and Quartz Sand Area

**Figure 14:**
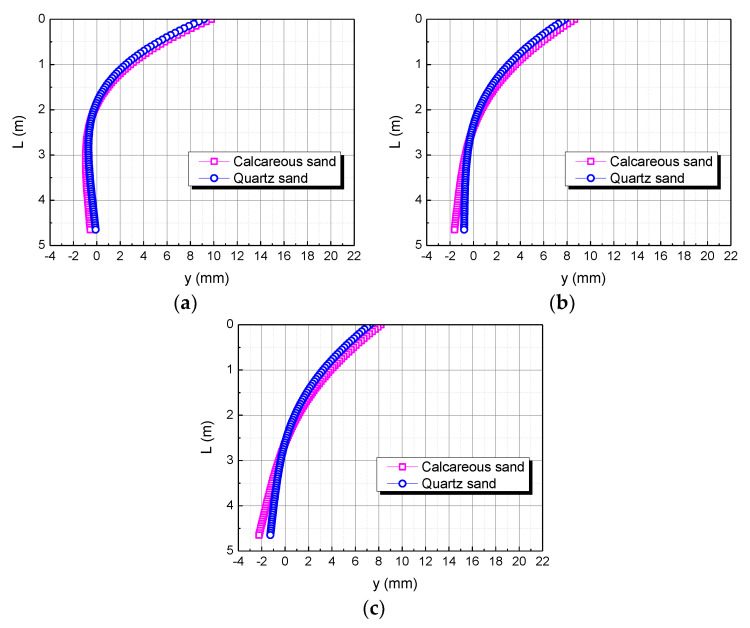
Effect of elastic modulus of pile on horizontal displacement of pile. (**a**) E; (**b**) 2E; (**c**) 3E.

The elastic modulus of the pile body is taken as E, 2E, and 3E, respectively. The influence of the elastic modulus of the pile body on the horizontal displacement of the pile body is shown in Figure 14. When the elastic modulus of the pile body is E, the horizontal displacement of the pile top in the calcareous sand area is 0.63122 mm larger than in the quartz sand area. When the elastic modulus of the pile body is 2E, the horizontal displacement of the pile top in the calcareous sand area is 0.80400 mm larger than in the quartz sand area. When the elastic modulus of the pile body is 3E, the horizontal displacement of the pile top in the calcareous sand area is 0.92964 mm larger than in the quartz sand area. The greater the elastic modulus of the pile body, the greater the difference between the two. It may be because the greater the elastic modulus of the pile body, the greater the stiffness of the pile body, which makes calcareous sand easier to compress than quartz sand.

### 4.4. Comparison of Horizontal Load in Calcareous Sand and Quartz Sand Area

**Figure 15:**
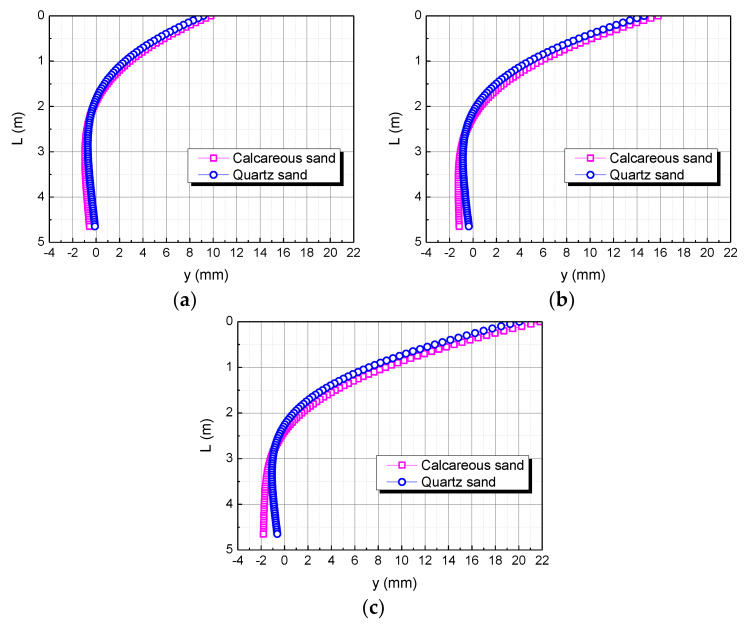
Effect of horizontal load on horizontal displacement of pile. (**a**) H; (**b**) 2H; (**c**) 3H.

The horizontal load is taken as H, 2H, and 3H, respectively. The influence of the horizontal load on the horizontal displacement of the pile body is shown in Figure 15. It can be seen that when the horizontal load is H, the horizontal displacement of the pile top of the foundation in the calcareous sand area is 0.63122 mm larger than in the quartz sand area. When the horizontal load is 2H, the horizontal displacement of the pile top in the calcareous sand area is 1.20301 mm larger than in quartz sand area. When the horizontal load is 3H, the horizontal displacement of the pile top in the calcareous sand area is 1.77480 mm larger than in the quartz sand area. The greater the horizontal load, the greater the difference between the two. It may be because the greater the horizontal load, the greater the force per unit area, which makes calcareous sand easier to compress than quartz sand.

### 4.5. Comparison of Bending Moment in Calcareous Sand and Quartz Sand Area

**Figure 16:**
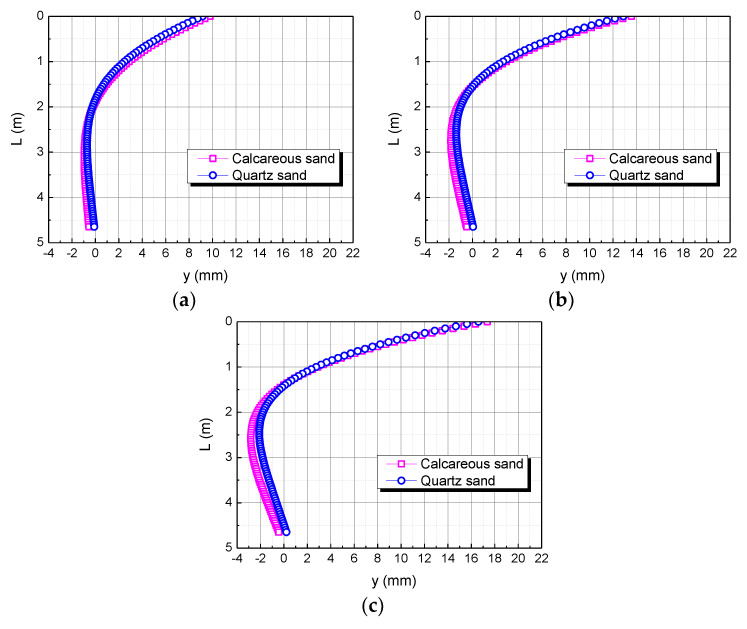
Effect of bending moment on horizontal displacement of pile. (**a**) M; (**b**) 2M; (**c**) 3M.

The bending moment is taken as M, 2M, and 3M, respectively. The influence of the bending moment on the horizontal displacement of the pile body is shown in Figure 16. When the bending moment is M, the horizontal displacement of the pile top of the pile foundation in the calcareous sand area is 0.63122 mm larger than in quartz sand area. When the bending moment is 2M, the horizontal displacement of the pile top in the calcareous sand area is 0.69065 mm larger than in the quartz sand area. When the bending moment is 3M, the horizontal displacement of the pile top in the calcareous sand area is 0.75007 mm larger than in the quartz sand area. The greater the bending moment, the greater the difference between the two. It may be because the greater the bending moment, the greater the force per unit area, which makes calcareous sand easier to compress than quartz sand.

### 4.6. Comparison of Void Ratio in Calcareous Sand and Quartz Sand Area

**Figure 17:**
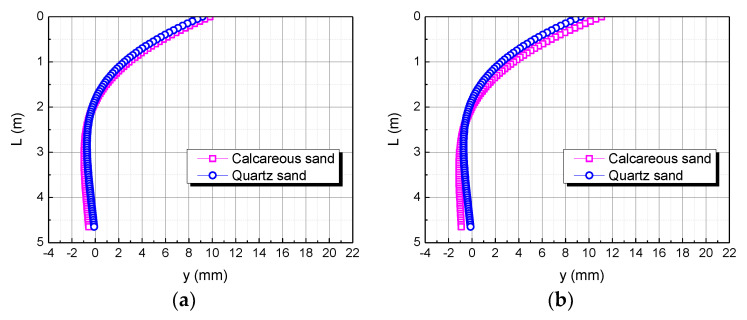

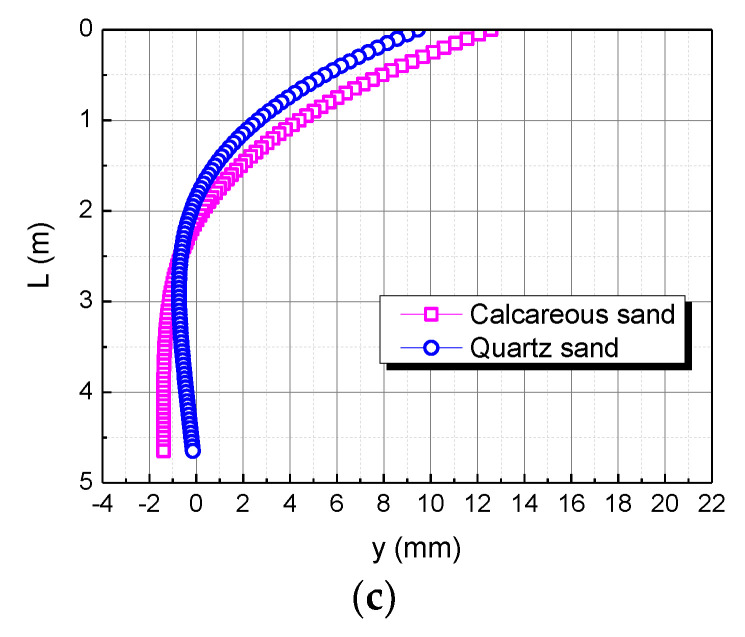
Effect of void ratio on horizontal displacement of pile. (**a**) e; (**b**) 1.1e; (**c**) 1.2e.

The void ratio is taken as e, 1.1e, and 1.2e, respectively, the adopted void ratio e of calcareous sand is 0.998 and that of quartz sand is 0.4, corresponding to an identical relative density of 80%. The influence of the void ratio on the horizontal displacement of the pile body is shown in Figure 17. When the void ratio is e, the horizontal displacement of the pile top in the calcareous sand area is 0.63122 mm larger than in the quartz sand area. When the void ratio is 1.1e, the horizontal displacement of the pile top in the calcareous sand area is 1.77762 mm larger than in the quartz sand area. When the void ratio is 1.2e, the horizontal displacement of the pile top in the calcareous sand area is 3.1107 mm larger than in the quartz sand area. The greater the void ratio, the greater the difference between the two. This may be due to the fact that calcareous sand is more easily compressed than quartz sand due to the greater void ratio.

In general, under the same conditions, the horizontal displacement of the pile top in the calcareous sand area is greater than that in the quartz sand area. It may be due to the compressive modulus of calcareous sand being less than that of quartz sand at the same depth, which makes calcareous sand easier to compress than quartz sand.

The studies of Coop et al. (2004) and Shen Yang et al. (2019) showed that calcareous sand can be mechanically broken under a lower external load than quartz sand, including breaking, crushing, and grinding of particles; this phenomenon can reasonably explain the compressive modulus of calcareous sand being less than that of quartz sand, which makes the horizontal displacement of the pile top in the calcareous sand area greater than that in the quartz sand area under the same conditions [34,35].

## 5. Conclusions

(1) Through case verification, the calculation results of the Pasternak foundation model are closer to the observed results than the Winkler foundation model. In addition, Based on the Winkler foundation model, the Pasternak foundation model assumes that there is a shear layer on the spring element, and this layer can only produce shear deformation but not compressible deformation; it is more in line with engineering practice. Therefore, we recommend the Pasternak foundation model while designing a pile foundation in calcareous sand area;

(2) The compressive modulus of calcareous sand was discovered to increase nonlinearly with the increase of buried depth. When the confining pressure is larger, the growth of the compressive modulus gradually slows down. When the confining pressure is 400 kPa, the compressive modulus of calcareous sand is 21.6 Mpa; when the confining pressure is 800 kPa, the compressive modulus of calcareous sand is only 28.3 Mpa;

(3) Under the same confining pressure, the compressive modulus of calcareous sand is smaller than that of quartz sand. The larger the confining pressure, the larger the difference between the two. When the confining pressure is 25 kPa, the compressive modulus of calcareous sand is 8.2 Mpa, the compressive modulus of quartz sand is 9.2 Mpa; when the confining pressure is 1600 kPa, the compressive modulus of calcareous sand is 38.5 Mpa, but the compressive modulus of quartz sand is as high as 83.5 Mpa;

(4) In this study, the pile length has little effect on the horizontal displacement of the pile top in the calcareous sand area. In addition, as the pile diameter increases, the elastic modulus of the pile increases, the horizontal load decreases, the bending moment decreases, and the void ratio decreases, the horizontal displacement of the pile top decreases. Therefore, when designing horizontally loaded piles in the calcareous sand area, in order to control the horizontal displacement of the pile top, a large diameter and a high modulus pile foundation can be selected, the horizontal load and bending moment of the pile foundation can also be controlled, and a calcareous sand area with a small void ratio can be selected for construction as much as possible;

(5) Under the same conditions, the horizontal displacement of the pile top in calcareous sand area is greater than in the quartz sand area. This is mainly due to the fact that the compression modulus of calcareous sand is smaller than that of quartz sand under the same conditions, making calcareous sand easier to compress than quartz sand. Calcareous sand can be mechanically broken under a lower external load than quartz sand can effectively explain the above phenomenon.

## Data Availability

The data presented in this study are available on request from the corresponding author.
